# Co-field-reconciled direct growth of 6-inch monolayer graphene

**DOI:** 10.1093/nsr/nwaf562

**Published:** 2025-12-15

**Authors:** Feifan Liu, Li Jia, Aoran Li, Yinghan Li, Wenze Wei, Yuanyuan Qiu, Ziang Chen, Kaixuan Zhou, Ting Cheng, Qingqing Ji, Zhongfan Liu, Jingyu Sun

**Affiliations:** College of Energy, Soochow Institute for Energy and Materials Innovations, Jiangsu Key Laboratory of Advanced Negative Carbon Technologies, Soochow University, Suzhou 215006, China; Beijing Graphene Institute, Beijing 100095, China; Beijing Graphene Institute, Beijing 100095, China; School of Physical Science and Technology, ShanghaiTech University, Shanghai 201210, China; College of Energy, Soochow Institute for Energy and Materials Innovations, Jiangsu Key Laboratory of Advanced Negative Carbon Technologies, Soochow University, Suzhou 215006, China; Beijing Graphene Institute, Beijing 100095, China; College of Energy, Soochow Institute for Energy and Materials Innovations, Jiangsu Key Laboratory of Advanced Negative Carbon Technologies, Soochow University, Suzhou 215006, China; Beijing Graphene Institute, Beijing 100095, China; School of Physical Science and Technology, ShanghaiTech University, Shanghai 201210, China; College of Energy, Soochow Institute for Energy and Materials Innovations, Jiangsu Key Laboratory of Advanced Negative Carbon Technologies, Soochow University, Suzhou 215006, China; Beijing Graphene Institute, Beijing 100095, China; Department of Chemistry, City University of Hong Kong, Hong Kong 999077, China; School of Physical Science and Technology, ShanghaiTech University, Shanghai 201210, China; Beijing Graphene Institute, Beijing 100095, China; College of Energy, Soochow Institute for Energy and Materials Innovations, Jiangsu Key Laboratory of Advanced Negative Carbon Technologies, Soochow University, Suzhou 215006, China; Beijing Graphene Institute, Beijing 100095, China

**Keywords:** graphene, 6-inch wafer, direct growth, batch uniformity, co-field optimization

## Abstract

The transfer-free synthesis of inch-scale high-quality graphene on insulators is of paramount importance for emerging electronic and optoelectronic applications. Nevertheless, recent efforts at direct growth via the chemical-vapor-deposition route failed to produce monolayer graphene with a large wafer size (i.e. 6 inches) affording scalable uniformity and batch repeatability. Here, we report a co-field-reconciled synthetic strategy in which the synergistic optimization of thermal and gas-flow fields readily allows the uniform growth of 6-inch monolayer graphene over a sapphire wafer with batch-production capability. The temperature and flow fields are dictated via the concurrent deployment of a graphite gasket and a gas distributor plate, with the effectiveness evidenced by simulation and wafer-level characterization results. Theoretical calculations reveal that our route lowers the methane-decomposition barrier and restrains multilayer nucleation. The thus-prepared graphene exhibits impressive crystal quality, spatial uniformity and electrical performance. Six-inch wafer-scale top-gated graphene field-effect transistor arrays showcase the consistent device characteristics, with a room-temperature mobility average rivaling the state-of-the-art examples. The generality of such a route could be extended to other insulating substrates, including SiC, WC, Si_3_N_4_ and SiO_2_. This work achieves co-field optimization during wafer-level graphene growth over insulators and lays the foundation for advancing the large-scale integration of graphene.

## Teaser

This work reports the co-optimization of temperature and flow fields in a cold-wall chemical vapor deposition reactor, allowing monolayer graphene growth directly over 6-inch sapphire substrate toward wafer-level device integration.

## INTRODUCTION

Graphene has stimulated extensive research interest for emerging electronic and optoelectronic device applications owing to its unique properties [1–5]. Synthesis determines the future. A central requirement for translating its full potential into practical technologies lies in the controllable production of wafer-scale graphene affording exceptional film uniformity [6]. Recent years have witnessed the mature demonstration of graphene wafer synthesis by employing chemical vapor deposition (CVD) routes over metals such as Cu [7–11]. In this sense, a tedious and costly transfer procedure is unavoidable, which normally induces cracks, contamination and foreign doping into the graphene, thereby degrading the film uniformity and device reliability, especially at a wafer level [12].

To circumvent the issues associated with the transfer, the direct synthesis of graphene on target substrates has been deemed a promising alternative strategy [13–18]. Pioneer work to obtain high-quality graphene on SiC was exemplified by inducing silicon sublimation from SiC at an elevated temperature of ∼1600°C [19,20], yet the high cost of SiC and limited space for synthetic apparatus dampen its large-scale wafer (i.e. 6-inch) production. C-plane sapphire is an economical insulator that combines excellent thermal stability, hexagonal lattice symmetry and well-established manufacture capability [21]. It has been demonstrated as an ideal support for growing 2D materials including graphene and transition-metal dichalcogenide [22–24]. As for graphene, a single-crystal monolayer at a size of 2 inches was realized at a Al_2_O_3_(0001)/Cu(111) interface through a multi-cycle plasma etching-assisted CVD route within a hot-wall furnace [25]. However, such synthesis requires complex procedures with post Cu delamination, which might not be favorable for scaling up. A cold-wall approach otherwise offers the compelling advantages of process simplicity and time-saving growth [26]. Fig. S1 shows a direct comparison of the growth processes between cold-wall and hot-wall CVD systems with respect to preparing transfer-free graphene/sapphire wafers. It is evident that the cold-wall CVD exhibits significantly enhanced system heating and graphene growth rates, thereby substantially improving production efficiency. Our previous endeavor dealt with the homogeneous fabrication of monolayer graphene over sapphire at a temperature of 1400°C by using cold-wall CVD. Unfortunately, the production of graphene was limited to a maximum wafer size of 2 inches [27].

Inspired by these considerations, we report herein the batch production of monolayer graphene with high uniformity and quality at a 6-inch wafer scale on single-crystal c-plane sapphire substrates. This is achieved by the systematic operational dictation of thermal and flow-field distributions in a homemade cold-wall CVD reactor, in which a graphite gasket is employed to homogenize the substrate surface temperature and the gas distributor plate is modified to eliminate the stagnation flow zone. Our computational fluid dynamics (CFD) simulations indicate a marked improvement in the thermal and flow-field distributions at the substrate, which are corroborated by the sheet-resistance and optical transmittance mapping comparisons before and after optimization. Theoretical calculation via molecular dynamics implies that the energy barrier of CH_4_ decomposition into CH_3_ in our cold-wall growth mode (1350°C) is reduced by 0.5 eV compared with that of the typical hot-wall mode (1300°C), accompanied by the increased reaction speed for ∼40 times. The cold-wall growth is beneficial for avoiding the generation of complex gas-phase species and multilayer island formation, confining itself to a bare surface reaction to render monolayer graphene. These 6-inch wafer-scale graphene monolayers are verified to exhibit impressive crystallinity and uniformity through exhaustive instrumental characterizations. In addition, graphene field-effect transistor (GFET) arrays with an industry-compatible top-gate configuration are fabricated on a 6-inch wafer, exhibiting highly homogeneous electrical characteristics.

## RESULTS AND DISCUSSION

### Six-inch graphene wafer production

**Figure 1.**
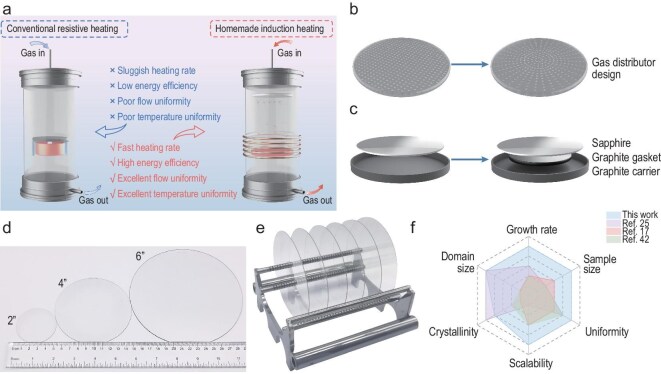
Direct growth of monolayer graphene on 6-inch sapphire wafers. (a) Schematic comparison of cold-wall CVD systems: conventional resistive heating (left panel) and our homemade induction heating (right panel). (b) Schematic illustration of the optimized gas distributor plate. (c) Schematic showing the modified graphite heater. (d) Digital photograph of as-synthesized 2-, 4-, and 6-inch monolayer graphene/sapphire wafers. (e) Digital photograph showing the batch production of 6-inch monolayer graphene/sapphire wafers. (f) Radar-chart comparison of our growth route and other direct CVD approaches for graphene synthesis.

In terms of a conventional cold-wall CVD reactor, heat is exclusively supplied via radiative and conductive transfer from a resistive heating element (Fig. 1a, left panel) [28]. Such a setup might result in sluggish heating rates, low energy efficiency and substantial nonuniformity in both the thermal and the gas-flow fields, which are not conducive to large-area graphene synthesis [29–31]. Our homemade electromagnetic induction-heated cold-wall reactor effectively mitigates these limitations: it directly induces eddy-current Joule heating within the graphite carrier through an alternating magnetic field, implementing an efficient ‘carrier self-heating’ mode. This would allow a 30%–50% increase in the heating rate as compared with the resistive heating mode. In parallel, the introduction of a gas distributor plate ensures the homogeneous distribution of the carbon gaseous precursor across the substrate surface—a key prerequisite for uniform graphene nucleation and growth (Fig. 1a, right panel).

As the size of the wafer substrate increases, realizing uniform flow and temperature fields becomes essential to sustain the wafer-level film quality. To regulate the gas flow over the substrate, the opening hole pattern of the gas distributor plate is optimized, as depicted in Fig. 1b. As for the rectangular-array configuration, the plate tends to generate a stagnation zone near the center of the substrate, giving rise to inhomogeneous flow across the wafer surface. In contrast, the updated circular-array pattern helps to eliminate perforations in the central region, effectively suppressing flow stagnation and hence improving flow uniformity.

It is worth noting that the skin effect would cause eddy currents to concentrate predominantly within the edge regime of the graphite carrier during electromagnetic induction heating. According to Joule’s Law, the heat-generation rate is proportional to the square of the current density [29]. Therefore, the edge regime of the carrier initiates more heat over the inner region, resulting in deviated temperature distribution. As the wafer size scales up, the associated temperature-gradient effect becomes markedly pronounced. To dictate the thermal field, a graphite gasket is introduced in between the growth substrate and the graphite carrier (Fig. 1c). In such a manner, the central regime of the wafer is subject to conductive heating via the graphite gasket, while the edge regime is heated by radiation and convection. Our modification effectively addresses the temperature gradient caused by the skin effect.

In a typical synthesis trial, a 6-inch c-plane sapphire wafer substrate is placed onto the modified graphite carrier. The growth chamber is maintained at a pressure of 3000 Pa under an Ar/H_2_ atmosphere, while the temperature is ramped to ∼1400°C and stabilized for 10 min. Subsequently, CH_4_ is introduced to initiate the graphene formation, followed by cooling down to room temperature in Ar/H_2_ upon growth (Fig. S2). Accordingly, 6-inch graphene/sapphire wafers could be produced. Figure 1d comparatively presents our directly grown graphene/sapphire wafer samples affording different wafer sizes (2, 4 or 6 inches). To evaluate the reproducibility of our method, sheet-resistance (*R*_s_) measurements were carried out on representative samples from five adjacent batches, with each batch showcasing an average *R*_s_ value of <650 Ω sq^−1^ (Fig. S3). In addition, the optical transmittance spectrum exhibits a transmittance of ∼97.7% at 550 nm, implying the monolayer feature of the synthesized graphene film (Fig. S4). The digital photograph shown in Fig. 1e shows the batch-produced 6-inch graphene wafers created by using our strategy. A radar performance map draws a comparison between our approach and representative methods, revealing superior growth rate, wafer size and film uniformity, together with competitive crystallinity, domain size and product scalability (Fig. 1f and Table S1).

### Co-field regulation at a 6-inch wafer scale

**Figure 2.**
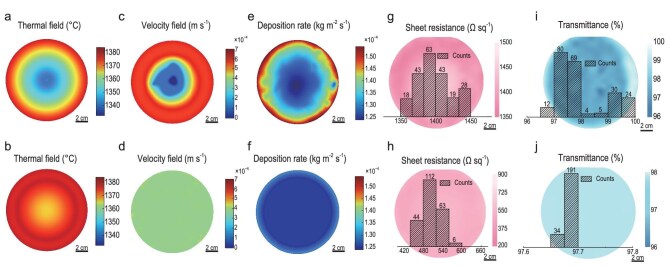
Regulation of thermal and flow fields for uniform growth of graphene on 6-inch sapphire wafers. (a, c, e) Simulated distributions of (a) temperature, (c) velocity and (e) deposition rate on the substrate surface before optimization. (b, d, f) Simulated distributions of (b) temperature, (d) velocity and (f) deposition rate on the substrate surface after optimization. Spatial mapping of (g) sheet resistance and (i) optical transmittance with corresponding statistical distributions for the 6-inch graphene/sapphire wafers before optimization. Spatial mapping of (h) sheet resistance and (j) optical transmittance with corresponding statistical distributions for the 6-inch monolayer graphene/sapphire wafers after optimization.

The effectiveness of the co-field regulation strategy was evaluated through CFD simulations. Figure 2a displays the temperature distribution across the substrate surface during graphene growth, clearly showing a gradual decrease from the edge to the center. This thermal profile is in good agreement with the Multiphysics analysis (Fig. S5). In detail, Fig. S5a illustrates the electromagnetic induction density distribution, highlighting the spatial nonuniformity of the electromagnetic field during heating. Figure S5b shows the isotherm contours, further demonstrating the gradient feature of the temperature field (Movie S1). The actual temperature value over the substrate region was recorded, revealing a maximum temperature difference of ∼50°C (Fig. S6).

The introduction of the graphite gasket essentially alters the wafer-heating behavior, leading to a notable improvement in thermal field uniformity (Fig. S7). As shown in Fig. S8, the distribution of isothermal gradient lines along the radial direction becomes sparser, indicative of a clear enhancement of the temperature homogeneity. A dynamic demonstration of this heating process is shown in Movie S2. To probe the gasket-size impact upon the substrate temperature distribution, simulations were carried out by employing gaskets ranging in diameter from 80 to 130 mm (Figs S9 and S10). The results suggest that a size of 110 mm delivers the optimized performance, with a maximum in-plane temperature difference of merely 5°C. Figure 2b displays the simulated temperature distribution on the substrate surface with the applied gasket, showing a substantial reduction in the thermal gradient with improved temperature uniformity (Fig. S11). Furthermore, under our experimental conditions, the graphite gasket contributes negligibly to the gas-phase carbon and has no substantial influence on the graphene growth (Fig. S12).

Figure 2c shows the gas velocity distribution when using a typical gas distributor plate with a rectangular-array pattern. A pronounced stagnation region, where the velocity approaches zero, forms at the substrate center. Meanwhile, the velocity remains relatively high near the edge, resulting in a maximum velocity difference of 7 × 10^−4^ m·s^−1^. Under these thermal and flow conditions, the simulated deposition of active carbon species exhibits a distinct edge–center disparity (Fig. 2e). The deposition rate is lowest in the central region (∼1.25 × 10^−4^ kg·m^−2^·s^−1^) and highest over the edges (up to ∼1.54 × 10^−4^ kg·m^−2^·s^−1^). Such nonuniformity could be attributed to the fact that the central region suffers from limited mass transport and lower surface temperatures. Figure 2d illustrates the gas distribution over the substrate when employing a modified gas distributor plate with a circular-array pattern. The gas velocity at the center reaches ∼3.5 × 10^−4^ m·s^−1^, effectively eliminating the stagnation zone. Concurrently, the velocity at the edge region is reduced, leading to a uniform flow field. Subsequent simulations of the deposition rate following co-field regulation are presented in Fig. 2f, which reveals a maximum in-plane variation of as small as 0.06 × 10^−4^ kg·m^−2^·s^−1^.

It is noted that previous wafer-scale CVD synthesis studies usually dealt with either flow/precursor homogenization in a hot-wall setup or cold-wall growth without field optimization (Table S2). In contrast, our co-field-reconciled strategy simultaneously flattens the in-plane temperature gradient and homogenizes the gas flow, so that the local carbon flux and mass transport are uniformly distributed across the 6-inch insulating wafers. To gain an insight into the effectiveness of such a strategy, the electrical conductivity and optical transmittance of graphene films over the 6-inch wafer scale were characterized. Without optimization, the sheet-resistance map of a 6-inch graphene/sapphire wafer shows an average value of ∼1400 Ω sq^−1^ with significant spatial fluctuations (Fig. 2g). Similarly, the transmittance mapping shown in Fig. 2i exhibits pronounced variations, implying the detrimental impact of thermal and flow-field nonuniformity on film quality. Upon optimization, the sheet-resistance map presents an average value of ∼506 Ω sq^−1^, with the transmittance value centering at 97.7% (Fig. 2h and j), suggesting the production of a uniform graphene monolayer across all of the 6-inch wafers.

### Uniformity evaluation of the 6-inch wafer-scale graphene

**Figure 3.**
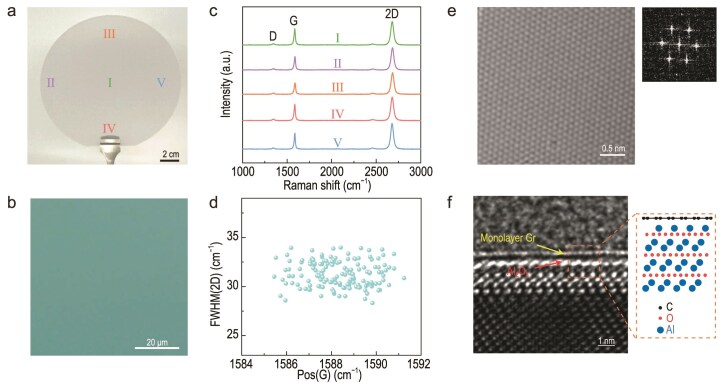
Characterizations of the as-grown monolayer graphene/sapphire wafers. (a) Photograph of a 6-inch monolayer graphene/sapphire wafer. (b) OM image of the as-grown graphene. (c) Typical Raman spectra collected from five representative positions from the graphene films synthesized directly on 6-inch monolayer graphene/sapphire wafer. (d) Statistical results of FWHM(2D) as a function of Pos(G). (e) Atomically resolved TEM image of the transferred monolayer graphene on a TEM grid. Inset: Fourier transform pattern. (f) Cross-sectional high-resolution TEM image of grown graphene on sapphire. Inset: interfacial O and Al atomic layers, as well as the graphene layer, are indicated in the orange-box region.

The elevated processing temperature (∼1400°C) in the cold-wall CVD system facilitates the decomposition of CH_4_ and enhances the transport of active carbon species [14]. Based on the co-field-reconciled growth strategy, homogeneous monolayer graphene could be readily attained on a 6-inch sapphire wafer in ∼30 min; Fig. 3a shows a digital photograph of a representative as-synthesized sample. The directly grown graphene forms a uniform and continuous film, exhibiting good optical contrast consistency without observable contaminants under optical microscopy (OM) (Fig. 3b).

The uniformity of the substrate surface morphology is crucial in realizing homogeneous graphene preparation. At temperatures of >1200°C, atomic steps emerge on the sapphire surface [32]. At higher annealing temperatures, both the width and the height of the steps gradually increase [33]. An in-plane temperature difference of ∼50°C exists within the wafer, leading to marked differences in step morphology between the edge and center regions (Fig. S13). Upon temperature-field optimization, the in-plane temperature uniformity of the wafer is significantly improved, resulting in consistent step width and height (Fig. S14). Atomic force microscopy characterizations of the step morphology in nine representative regions across the wafer manifest uniform morphology in all areas (Fig. S15).

Raman spectra collected from five different locations on the produced 6-inch graphene wafer are presented in Fig. 3c. All spectra display identical graphene features, with a G peak at 1587 cm^−1^, a 2D peak at 2680 cm^−1^ and an intensity ratio of the D and G peaks (*I*_D_/*I*_G_) of <0.1. The 2D peak is sharp and can be well fitted by using a single Lorentzian profile, with a high intensity ratio of the 2D and G peaks (*I*_2D_/*I*_G_) and a full width at half maximum (FWHM) of the 2D peak of ∼33 cm^−1^. These spectral features collectively confirm the formation of uniform, high-quality, adlayer-free monolayer graphene [34] via the co-field-reconciled strategy. In comparison, Raman spectra collected from five representative locations on the 6-inch graphene/sapphire wafer grown without optimization show that the values of *I*_D_/*I*_G_ and FWHM(2D) fall within the respective ranges of 0.4–0.6 and 33–38 cm^−1^ (Fig. S16), revealing pronounced variations in graphene quality across the entire wafer.

As shown in Fig. S17, Raman characterization was carried out at 30 randomly selected points across the optimized 6-inch graphene wafer. The collected spectra consistently show well-defined G and 2D peaks positioned at 1580 and 2680 cm^−1^, respectively, with no discernible D peak detected at 1350 cm^−1^. Accordingly, Raman mapping over a region of 10 μm × 10 μm was performed. Comparison of the *I*_2D_/*I*_G_ and *I*_D_/*I*_G_ maps before and after optimization reflects significant improvements in the signal consistency (Figs S18 and S19). These features confirm the formation of high-quality monolayer graphene [34] with exceptional spatial uniformity over the entire wafer.

Raman spectra were further collected from 100 randomly selected points on our optimized 6-inch graphene wafer. The 2D and G signals were fitted by using Lorentzian functions to extract their peak positions (Pos), FWHM and intensity/area ratios. Figure 3d shows the statistical distributions of Pos(G) = 1588 ± 1 cm^−1^ and FWHM(2D) = 31 ± 1 cm^−1^ (average ± 1 standard deviation). The consistency of the FWHM(2D) value implies that strain fluctuations within the graphene are at a fairly low level [35], corroborating the monolayer uniformity over the 6-inch wafer. In the meantime, other spectral parameters were obtained: FWHM(G) = 14 ± 2 cm^−1^, *A*_2D_/*A*_G_ (area ratio of the 2D and G peaks) = 4.3 ± 0.4, *I*_2D_/*I*_G_ = 1.7 ± 0.2 and Pos(2D) = 2679 ± 2 cm^−1^ (Fig. S20). Note that the correlation between Pos(2D) and Pos(G) could be well described by a linear fit with a slope of 1.16, indicating that strain effects are negligible [36–40]. Moreover, the grown graphene exhibits a low overall doping level, with the Fermi energy estimated at ∼170 meV [36,41]. As compared with previously reported results for direct graphene growth on insulators [13,14,16, 17, 32,42,43–47], these values suggest a distinct advantage, underscoring the high quality and scalability of the graphene achieved through co-field regulation.

To provide structural verification, the films were subsequently transferred onto transmission electron microscopy (TEM) grids. The atomically resolved TEM image and fast Fourier transform pattern shown in Fig. 3e demonstrate the high crystalline quality of the monolayer graphene. Cross-sectional TEM observations of the graphene/sapphire interface confirm the monolayer graphene growth directly on sapphire with continuous coverage (Fig. 3f and Fig. S21). Interestingly, the high-resolution image shown in Fig. 3f reveals the atomic structure of the graphene/sapphire interface, showing an Al-rich termination. Such a termination is expected because annealing at higher temperatures (>1200°C) under a low-pressure and reducing atmosphere leads to oxygen depletion at the sapphire surface [32]. This interfacial configuration is in sharp contrast to the O-rich termination typically observed in conventional CVD growth (at ∼1050°C) on sapphire [48]. In this sense, the presence of an Al-rich surface might be conducive to facilitating the decomposition of carbon precursors and hence promoting the graphene formation [27,32].

### Theoretical insights into monolayer formation

**Figure 4.**
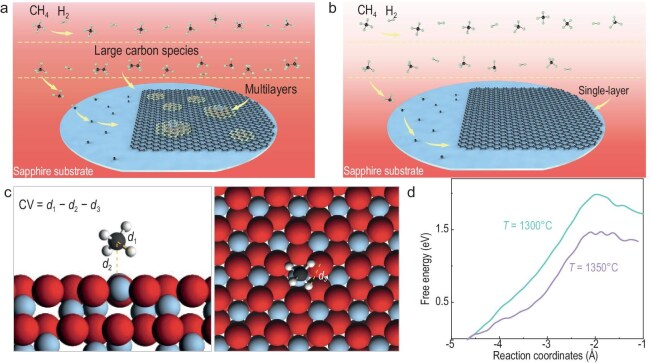
Theoretical insights via *ab initio* molecular dynamics simulations into the graphene monolayer formation. Schematic of reaction processes in the (a) hot-wall and (b) cold-wall CVD systems using CH_4_ and H_2_ as reactants. The background color indicates the environmental temperatures, with lighter colors representing lower temperatures. (c) Side view (left panel) and top view (right panel) of the initial structure and collective variable (CV) defined as the reaction coordinate. The O, Al, C and H atoms are represented by red, blue, black and white spheres, respectively. (d) Integrated free-energy profile along the reaction coordinate at different simulation temperatures.

When graphene is grown directly on insulators, the carbon-rich gaseous environment combined with the catalytically inert nature of the substrate surface generally favors multilayer formation rather than controlled monolayer production. As shown in Fig. 4a, in a conventional hot-wall CVD system, the relatively high-temperature region above the substrate promotes extensive gas-phase pyrolysis, in which CH_4_ molecules evolve into larger carbon species and clusters. These carbon clusters readily adsorb on the growing graphene surface and trigger secondary nucleation, resulting in the formation of contamination domains, which are mostly considered amorphous carbon. Furthermore, due to the intrinsically weak catalytic activity of sapphire, the surface diffusion of carbon species is limited and the graphene growth units are predominantly supplied from the gas phase rather than the substrate. This kinetic environment naturally leads to an island-like multilayer growth mode.

In stark contrast, our employed cold-wall CVD system establishes a fundamentally different growth regime in which the elevated temperature (∼1400°C) is merely confined to the substrate surface, significantly suppressing gas-phase reactions and the formation of large carbon clusters (Fig. 4b). Consequently, the dominant carbon source for graphene growth is derived from surface-mediated decomposition rather than uncontrollable gas-phase pyrolysis, enabling the formation of high-quality graphene monolayers.

To further understand the initial decomposition process of methane, we performed free-energy calculations based on *ab initio* molecular dynamics simulations (Fig. S22). Figure 4c displays the side and top views of the initial CH_4_ adsorption on the sapphire surface. The collective variable, defined as the reaction coordinate, is shown in the inset. Figure 4d presents the integrated free-energy profiles of CH_4_ decomposition, CH_4_ → CH_3_ + H, at different simulation temperatures (1300°C and 1350°C). At 1350°C, the reaction barrier is reduced by ∼0.5 eV compared with that at 1300°C, suggesting that CH_4_ dissociation at 1350°C proceeds ∼40 times (∼0.5 eV/*k*_B_*T*, where *k*_B_ is the Boltzmann constant) faster than that at 1300°C, in agreement with experimental observations that higher surface temperatures promote methane cracking and efficient carbon supply [27,49]. The combination of a confined surface-heating profile and uniform substrate temperature in our homemade cold-wall CVD system thus effectively accelerates CH_4_ decomposition and suppresses uncontrolled gas-phase reactions, providing a kinetic pathway to favor the tailored growth of high-quality monolayer graphene.

### Device performance based on the 6-inch graphene/sapphire wafer

**Figure 5.**
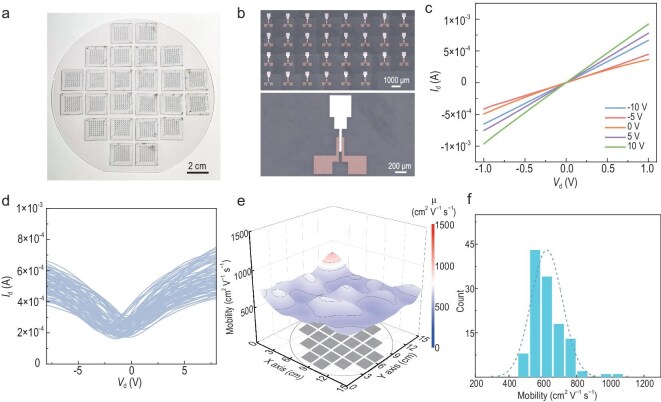
Electrical properties of 6-inch graphene wafers. (a) Photograph of top-gated graphene device arrays based on 6-inch sapphire wafer. (b) OM images of (top) TG-GFET array and (bottom) an individual device. (c) Output characteristic of a typical TG-GFET. (d) Transfer curves of arrayed graphene devices (*V*_ds_ = 1 V). (e) 3D color map showing the carrier mobility distribution of 60 TG-GFETs over the 6-inch graphene wafer. (f) Corresponding histogram and Gaussian fit of the mobilities for these graphene devices.

To facilitate the transfer-free construction of top-gated graphene field-effect transistor (TG-GFET) devices on the 6-inch wafers, each wafer was diced into 24 samples (2 × 2 cm^2^) for laboratory-compatible nanofabrication processes. As shown in Fig. 5a, TG-GFETs were fabricated in batches directly on these wafer samples, demonstrating the scalability of our device-fabrication technique. Figure 5b presents an optical microscopy image of the TG-GFET array with a magnified view of an individual device, featuring a graphene channel with both a length and a width of 50 μm, as well as a 50-nm-thick Al_2_O_3_ dielectric layer grown by using atomic layer deposition. The output and transfer characteristics of a representative TG-GFET device are provided in Fig. 5c and Fig. S23, respectively, showing the typical ambipolar field-effect modulation of graphene.

Subsequently, electrical measurements at room temperature and under ambient conditions were performed on the arrayed TG-GFET devices, with the source−drain voltage fixed at 1 V and the top-gate voltage swept from −10 to 10 V. The resulting transfer curves shown in Fig. 5d exhibit uniform electrical performance across all devices. Figure 5e displays the distribution of extracted electron/hole mobilities for another batch of 60 TG-GFETs randomly selected over the 6-inch wafer, in which the small fluctuation indicates high electrical uniformity of the graphene sample. A corresponding statistical histogram with a Gaussian fit of the field-effect mobilities is shown in Fig. 5f, yielding an average value of 624 ± 93 cm^2^ V^−1^ s^−1^, while the maximum mobility reaches ≤1014 cm^2^ V^−1^ s^−1^. Although the achieved mobility data do not yet rival the best performance of CVD graphene transferred from Cu substrates, these values are superior to the previously reported values for graphene directly grown on SiC, SiO_2_ or sapphire substrates [13,19,50–52] and evidently higher than that of the state-of-the-art wafer-level 2D semiconductors [31,53] (Table S3). Specifically, the direct-growth approach removes the need for complex large-area transfer and prevents polymer residues, metal contamination and mechanical damage [12]. It also yields a cleaner graphene/substrate interface and stronger adhesion, which are advantageous for subsequent device fabrication [25,32]. The current device performance indeed has room for improvement. The mobility enhancement remains limited by several factors, including the scattering induced by the top-gate dielectric [20], the absence of the four-probe exclusion of contact resistance, the relatively large channel dimensions (50 × 50 μm^2^) and the wafer-dicing technique. These collectively render it difficult to achieve high mobility values as compared with smaller device dimensions and/or grown substrate scales.

Electrical characterization at 83 K was further carried out to evaluate the device performances under cryogenic conditions. Fig. S24 compares the transfer characteristics of three representative devices at 298 and 83 K, showing improved electron–hole symmetry and field-effect mobilities at low temperatures, with the highest mobility value among them reaching 1200 cm^2^ V^−1^ s^−1^. Such electrical uniformity is a direct consequence of the reduced content of grain boundaries, which is attributed to the large crystal size and strong alignment of the grown graphene domains on the sapphire wafer [27]. This advancement might pave the way for the electronic and optoelectronic applications of graphene that typically necessitate insulating substrates. Moreover, our growth route is expected to be applicable to other insulating substrates, including SiC, WC, Si_3_N_4_ and SiO_2_ (Fig. S25).

## CONCLUSIONS

In summary, we present a co-field-reconciled strategy through the optimization of thermal and gas-flow fields, achieving the controlled growth of monolayer graphene with excellent spatial uniformity on 6-inch sapphire wafers. By incorporating a graphite gasket and optimizing the deflector plate design, the long-standing challenges of temperature gradients and flow stagnation in large-area growth within CVD systems are effectively overcome. Integrating theoretical simulations with experimental characterizations confirms the advantages of our approach in growth uniformity and reproducibility, readily establishing a scalable pathway to allow the production of 6-inch high-quality monolayer graphene. Furthermore, device-level evaluations verify impressive wafer-level uniformity. Our study offers a broadly applicable route toward the wafer-scale integration of 2D materials beyond graphene, providing a solid foundation for future advances in nanoelectronics and optoelectronics.

## METHODS

### CVD growth of graphene on 6-inch sapphire wafer

Graphene growth was carried out in a homemade cold-wall CVD system equipped with electromagnetic induction heating. The graphite carrier inside the chamber was heated by using an alternating magnetic field generated through the application of alternating current to the induction coil. The growth temperature was continuously monitored by using a thermocouple, whereas the chamber pressure was adjusted via closed-loop control. In the experiment, a commercial 6-inch c-plane sapphire substrate (purchased from Unionlight Technology Co. Ltd) was placed on the graphite carrier and the chamber was evacuated to remove residual air. Subsequently, H_2_ [500 standard cubic centimeters per minute (sccm)] and Ar (1000 sccm) were introduced into the chamber and distributed through the deflector plate. The substrate was then annealed by heating to ∼1400°C at a pressure of 3000 Pa for 10 min. After that, CH_4_ (100 sccm) was introduced as the carbon precursor and maintained for 30 min for graphene growth. Upon completion, the CH_4_ was shut off and the sample was cooled down naturally to room temperature under a mixed atmosphere of Ar and H_2_.

### Transfer of graphene

The transfer of graphene onto the TEM grid was achieved by using Polymethyl Methacrylate (PMMA) as an assisting agent. Initially, the graphene/sapphire sample was spin-coated with PMMA at a speed of 2000 rpm and then subjected to baking at 120°C for a duration of 15 min. Graphene film was detached from the sapphire substrate by immersing the sample in a 10 vol% NaOH solution. The resulting PMMA/graphene membrane was washed with deionized water and transferred onto TEM grids for drying under an infrared lamp. Subsequently, it was exposed to acetone vapor to dissolve the PMMA.

### Characterizations

The samples were characterized by using OM (Olympus DX51), Raman spectroscopy (RAMAN WITEC ALPHA300R; 532-nm laser excitation, 100 × objective lens), a four-probe electrical measurement system (CDE ResMap 178), TEM (FEI Tecnai F20, acceleration voltage at 200 kV), Atomic Force Microscope (Bruker Dimension Icon), a Ultraviolet–Visible (UV–Vis) spectrophotometer (Lambda 950) and a bench-top transmittance tester (SDR851).

### Device fabrication and measurement

Graphene device arrays were fabricated via a Microwriter UV litho-ACA (TuoTuo Technology Ltd), using a double-layer photoresist (LOR5A and S1805) for the graphene/sapphire wafer. Ni/Au (15 nm/20 nm) layers were deposited via thermal evaporation at a rate of 0.7 Å/s, following which a lift-off process was conducted to define the source/drain electrodes. The graphene channels were defined by using oxygen plasma etching (100 W, 50 sccm, 10^−5^ torr, 150 s, VISION 322 RIE) into regular shapes of 50 × 50 μm^2^. A 50-nm-thick Al_2_O_3_ gate dielectric layer was then deposited on the graphene channel at 200°C by using atomic layer deposition (Savannah S200). Finally, Ni/Au (15 nm/20 nm) gate electrodes were deposited by using thermal evaporation. Electrical measurements of the fabricated TG-GFETs were performed in an Instec probe station (HCP421VT-MPS) with a PRIMARIUS FS-Pro semiconductor parameter analyser.

## Supplementary Material

nwaf562_Supplemental_Files
