# Supplementary material for: Co-field-reconciled direct growth of 6-inch monolayer graphene
Source: Natl Sci Rev. 2025 Dec 15;13(3):nwaf562. doi: 10.1093/nsr/nwaf562 (PMC12866660; doi:10.1093/nsr/nwaf562)
Supplement: nwaf562_Supplemental_Files [file nwaf562_supplemental_files.zip › Supplementary data.pdf]

Supporting Information

Co-Field-Reconciled Direct Growth of 6-Inch Monolayer Graphene

Feifan Liu[1,2,†], Li Jia[2,†], Aoran Li[3,†], Yinghan Li[1,2], Wenze Wei[1,2], Yuanyuan Qiu[3], Ziang Chen[1], Kaixuan Zhou[2], Ting Cheng[4,*], Qingqing Ji[3,*], Zhongfan Liu[2,*] and Jingyu Sun[1,2,*]

[1]College of Energy, Soochow Institute for Energy and Materials Innovations, Jiangsu Key Laboratory of Advanced Negative Carbon Technologies, Soochow University, Suzhou 215006, China;
[2]Beijing Graphene Institute, Beijing 100095, China;
[3]School of Physical Science and Technology, ShanghaiTech University, Shanghai 201210, China;
[4]Department of Chemistry, City University of Hong Kong, Hong Kong 999077, China

***Corresponding authors**. E-mails: sunjy86@suda.edu.cn; zfliu@pku.edu.cn; jiqq@shanghaitech.edu.cn; ting.cheng@cityu.edu.hk
†Equally contributed to this work.

Supplementary Methods

CFD simulations

The computational fluid dynamics (CFD) simulations were performed in COMSOL Fluent based on the finite element method. The simulation model was developed to replicate the geometry of the homemade cold-wall CVD system, with representative parameters defined as follows: Ar flow rate of 1000 sccm, H_2 flow rate of 500 sccm, CH_4 flow rate of 100 sccm, chamber pressure of 3000 Pa, and the density, thermal conductivity, and specific heat capacity characteristics of the Al_2O_3 ceramic and sapphire substrate were assumed to 3.90 $g{\cdot}cm^{-3}$, 30 $W{\cdot}mK^{-1}$, 0.88 $J{\cdot}g^{-1}{\cdot}K^{-1}$ as well as 3.98 $g{\cdot}cm^{-3}$, 35 $W{\cdot}mK^{-1}$, 0.77 $J{\cdot}g^{-1}{\cdot}K^{-1}$.

Theoretical calculations

All first-principles calculations and *ab initio* molecular dynamics (AIMD) simulations were performed within the framework of density functional theory (DFT) as implemented in the Vienna ab initio simulation package (VASP) [1]. The interaction between valence electrons and ionic cores was described using the projector augmented-wave (PAW) method [2], while the exchange–correlation effects were treated using the generalized gradient approximation (GGA) with the Perdew–Burke–Ernzerhof (PBE) functional [3]. To improve the accuracy, spin polarization was included in all calculations, and the long-range van der Waals interactions were corrected using Grimme's DFT-D3 scheme [4]. The plane-wave cutoff energy was set to 400 eV, and structural optimizations were performed until the total energy converged to less than 10^{-5} eV and the residual force on each atom was below 0.01 eV/Å.

AIMD simulations were carried out in the canonical ensemble (NVT) using the Nosé–Hoover thermostat, with a time step of 0.5 fs and the Brillouin zone sampled at the Γ point. Given that sapphire is an inert oxide surface with negligible interaction with H_2, the presence

of H_2 is not expected to influence the CH_4 dissociation pathway or corresponding energy barrier. To this end, H_2 was not explicitly considered in our AIMD simulations. The substrate temperature was maintained at the target values (1300°C and 1350°C) to simulate the experimental environment. During conventional AIMD, the system typically remains trapped in local minima corresponding to metastable states, and transitions between minima are rare on the picosecond timescale, particularly when reaction barriers are high. To overcome this limitation, we adopted a slow-growth enhanced sampling AIMD approach (SG-AIMD) [5,6], in which constrained AIMD simulations were performed along a predefined collective variable (CV) corresponding to the methane dissociation pathway on the sapphire surface. Within this method, a small external bias is applied to drive the system quasistatically along the reaction coordinate under isothermal conditions, and the instantaneous mean forces are integrated using thermodynamic integration to obtain the free energy profile and corresponding free energy barriers. This approach allows us to accurately quantify the kinetic differences at various growth temperatures and directly correlate simulation results with experimental observations.

Supporting Figures and Tables

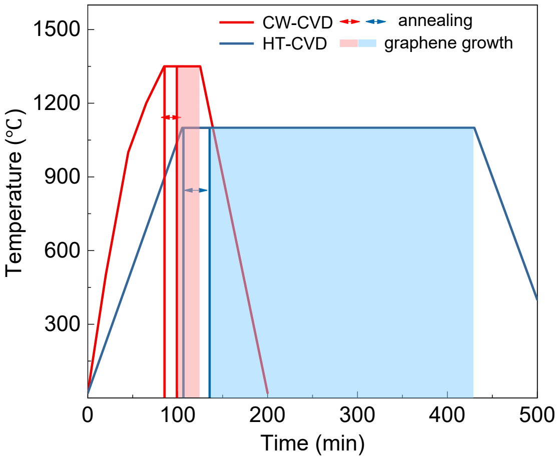

Figure S1. Comparison of temperature–time profiles for graphene growth on sapphire based on cold-wall (CW) and hot-wall (HT) CVD routes.

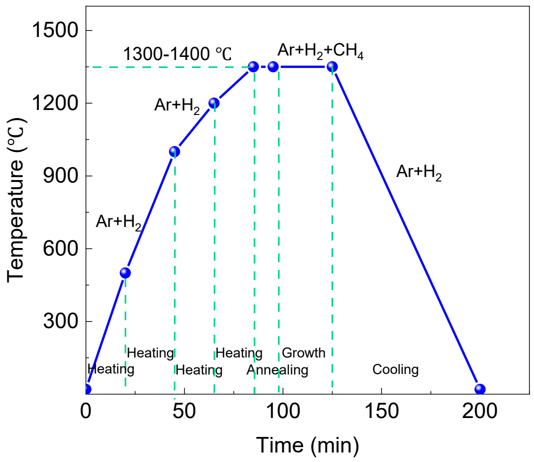

Figure S2. Temperature–time profile of the synthetic steps for producing 6-inch graphene/sapphire wafer by cold-wall CVD.

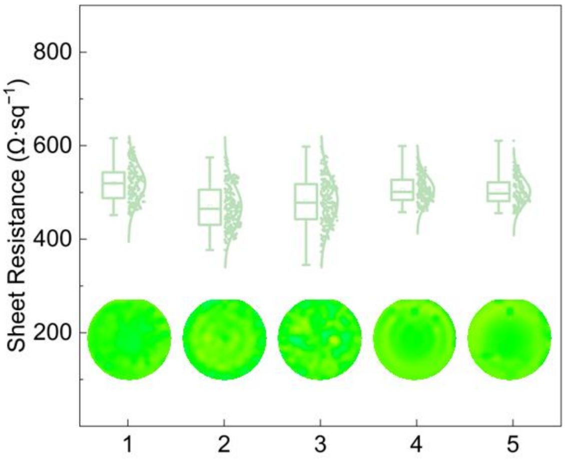

Figure S3. Sheet resistance mapping statistics of 5 representative 6-inch graphene/sapphire wafers from adjacent batches (with 225 data points per sample).

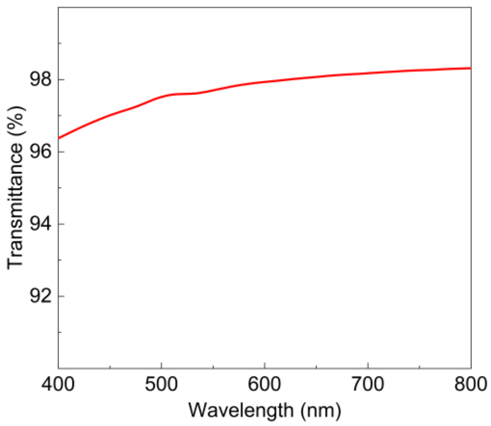

Figure S4. Optical transmittance spectrum of the as-prepared 6-inch graphene film.

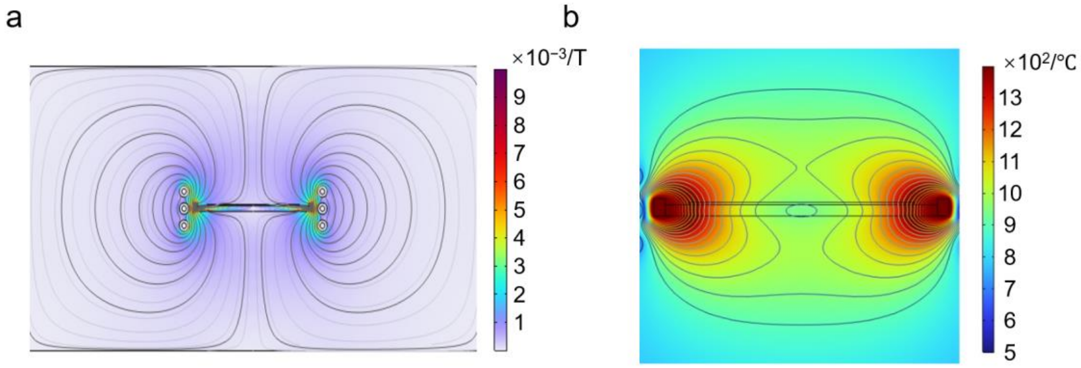

Figure S5. a) Electromagnetic induction density distribution and b) isotherm contours during electromagnetic induction heating without temperature field optimization.

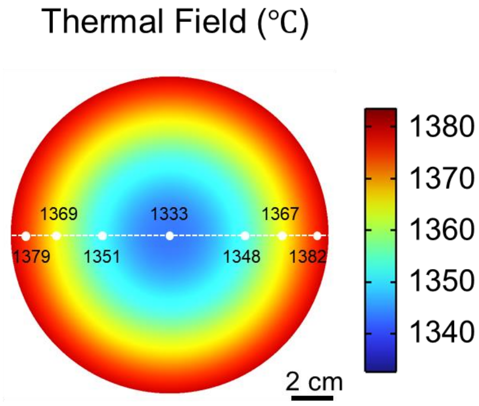

Figure S6. Spatial temperature distribution measured on the wafer surface without temperature field optimization.

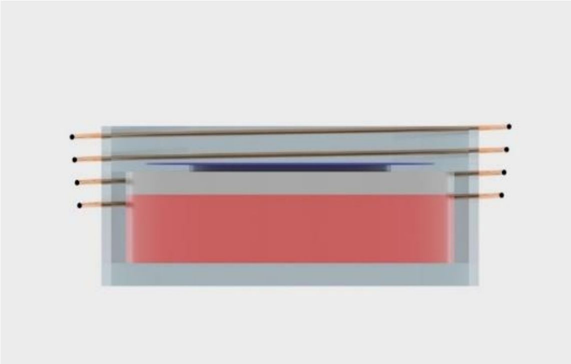

Figure S7. Schematic of electromagnetic induction-heated cold-wall CVD system by applying graphite gasket to improve thermal field uniformity.

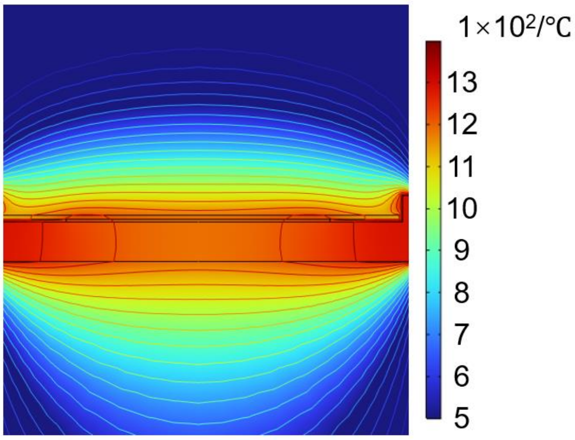

Figure S8. Isothermal line distribution with enhanced temperature homogeneity in the CVD reactor.

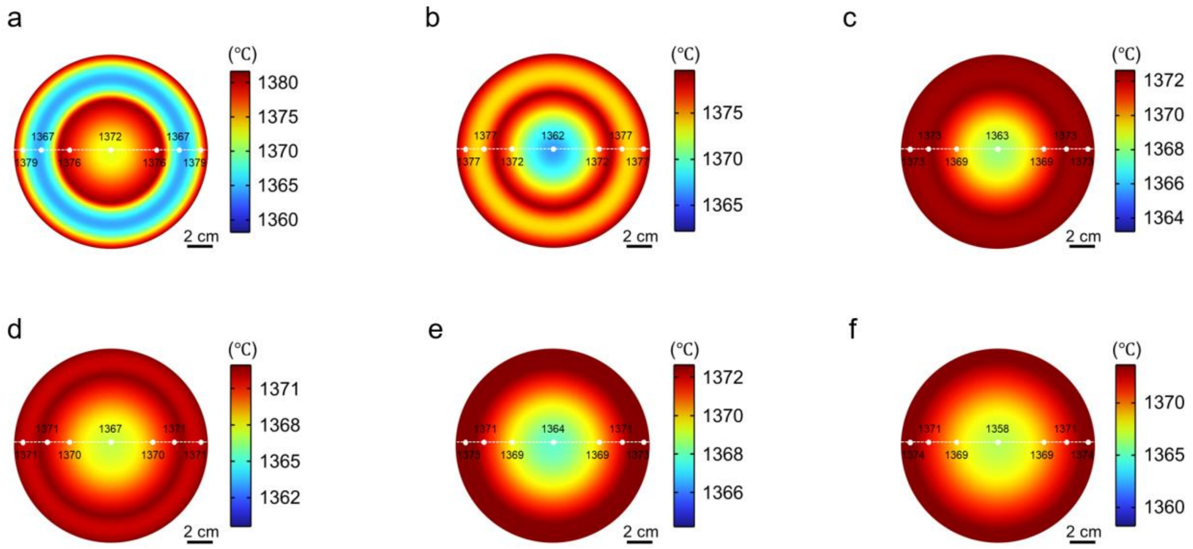

Figure S9. Substrate temperature distribution on the wafer surface with different graphite gasket diameters: a) 80 mm, b) 90 mm, c) 100 mm, d) 110 mm, e) 120 mm, and f) 130 mm.

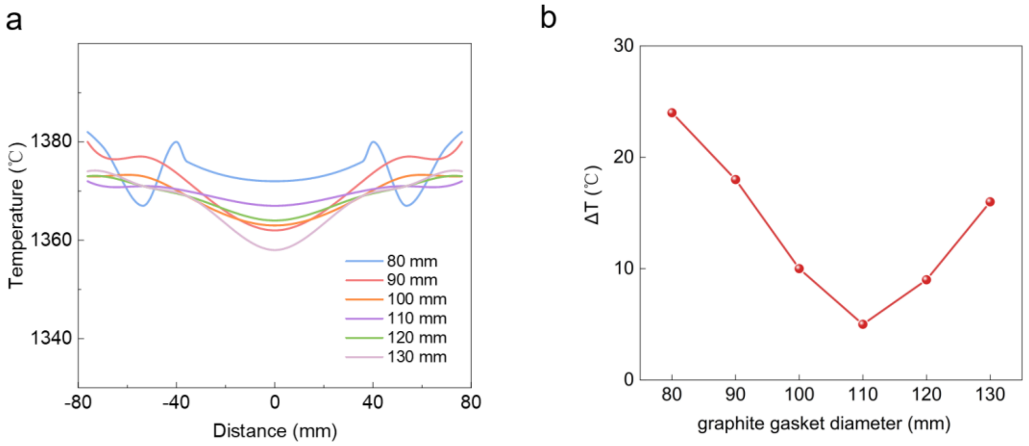

Figure S10. Statistical analysis of wafer surface temperature as a function of graphite gasket diameter. a) Radial temperature distribution across the wafer and b) maximum temperature difference for varied gasket diameters.

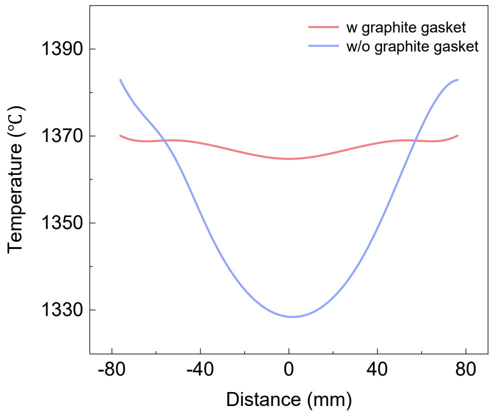

Figure S11. Comparison of radial temperature distribution on the wafer surface with/without the employment of graphite gasket.

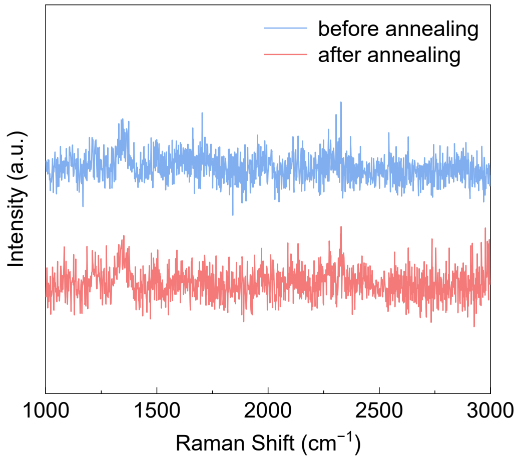

Figure S12. Raman spectra of the sapphire substrate before and after annealing for 30 min with the presence of graphite gasket.

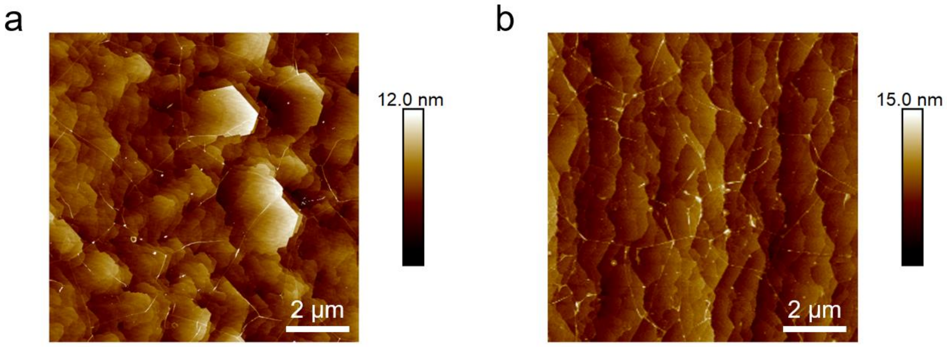

Figure S13. AFM images of graphene/sapphire wafer surface in different regions without thermal field optimization: a) edge region and b) central region.

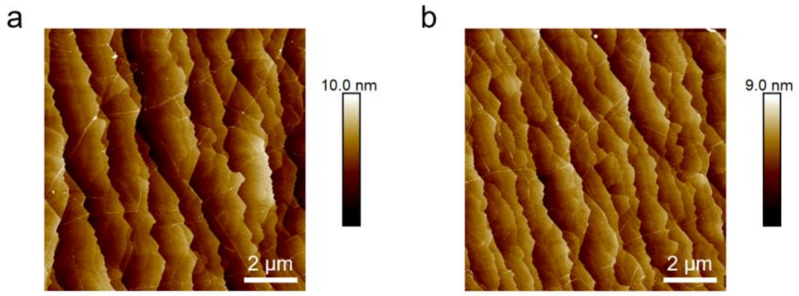

Figure S14. AFM images of graphene/sapphire wafer surface in different regions with thermal field optimization: a) edge region and b) central region.

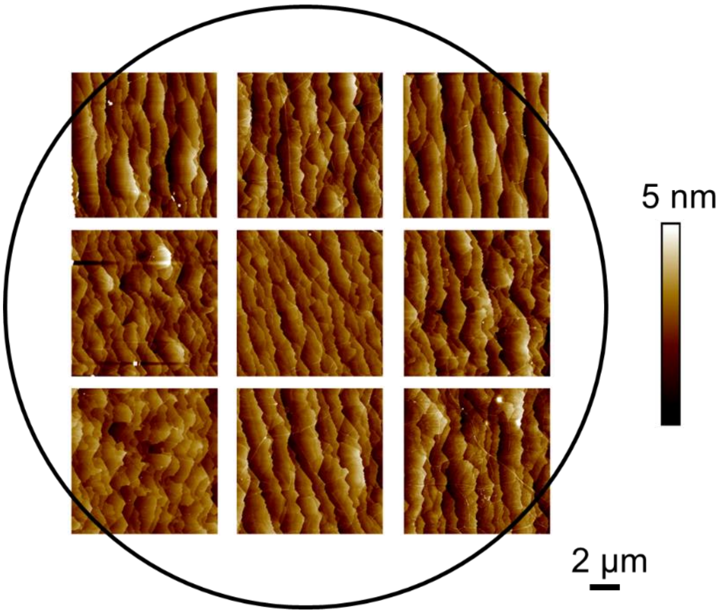

Figure S15. AFM images taken at 9 representative regions on 6-inch of graphene/sapphire wafer surface with thermal field optimization.

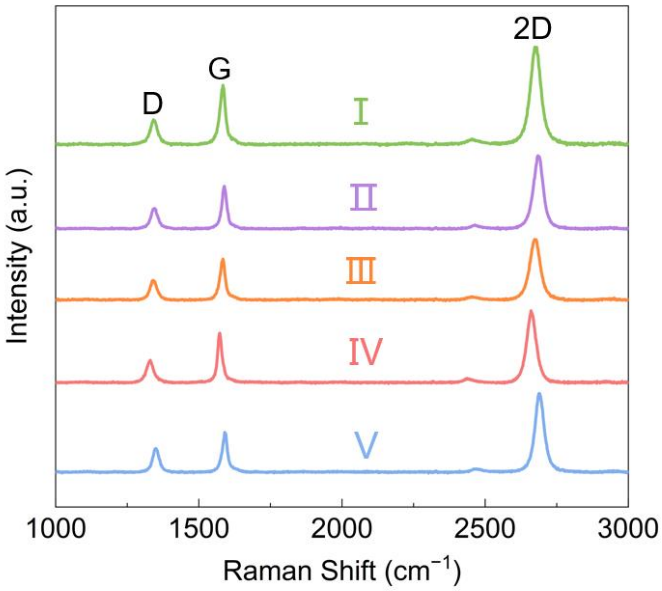

Figure S16. Raman spectra collected from 5 representative locations on the 6-inch graphene/sapphire wafer grown without field optimization.

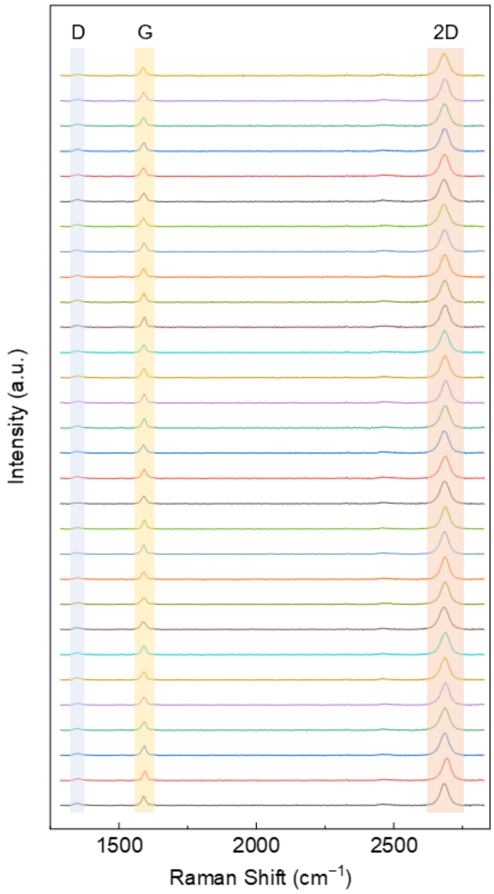

Figure S17. Raman characterization results at 30 randomly selected points across the optimized 6-inch graphene wafer.

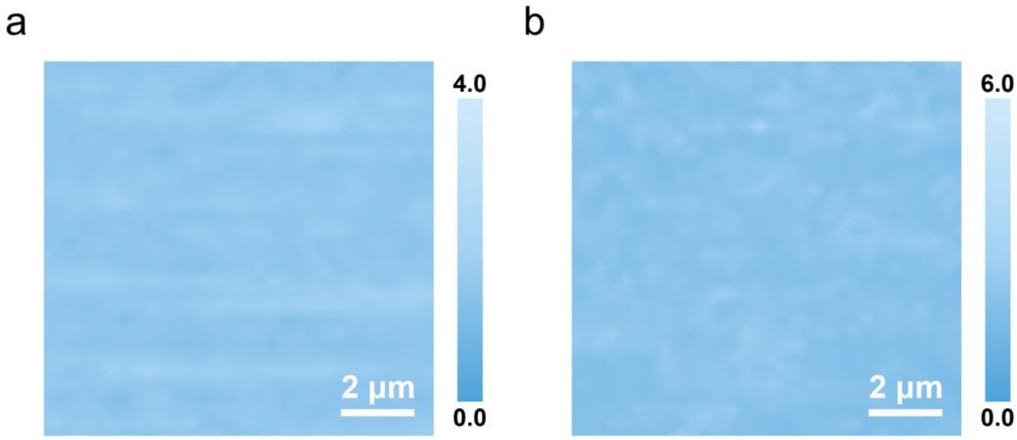

Figure S18. The comparison of I_{2D}/I_G maps a) without and b) with the field optimization.

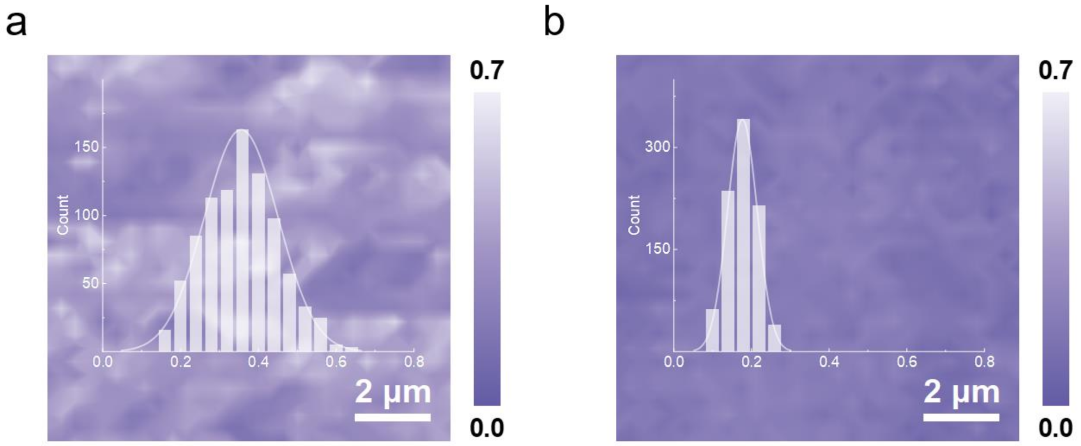

Figure S19. The comparison of I_D/I_G maps a) without and b) with the field optimization.

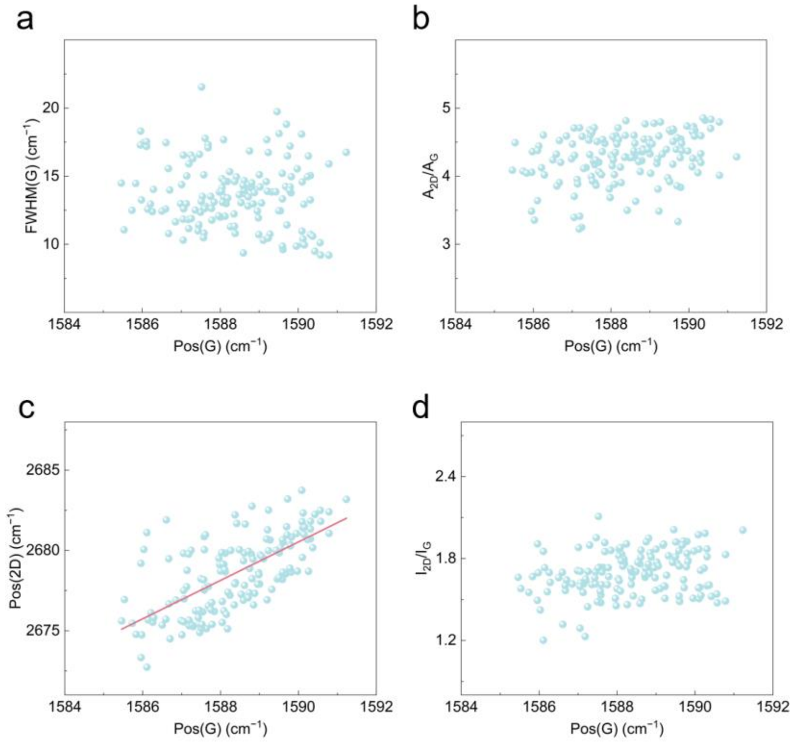

Figure S20. Statistical results of Raman spectra from 100 points on the 6-inch graphene/sapphire wafer: a) FWHM(2D) as a function of Pos(G), b) A_{2D}/A_G as a function of Pos(G), c) Pos(2D) as a function of Pos(G). The red line represents a linear fit to the data, with a slope of 1.16, d) I_{2D}/I_G as a function of Pos(G).

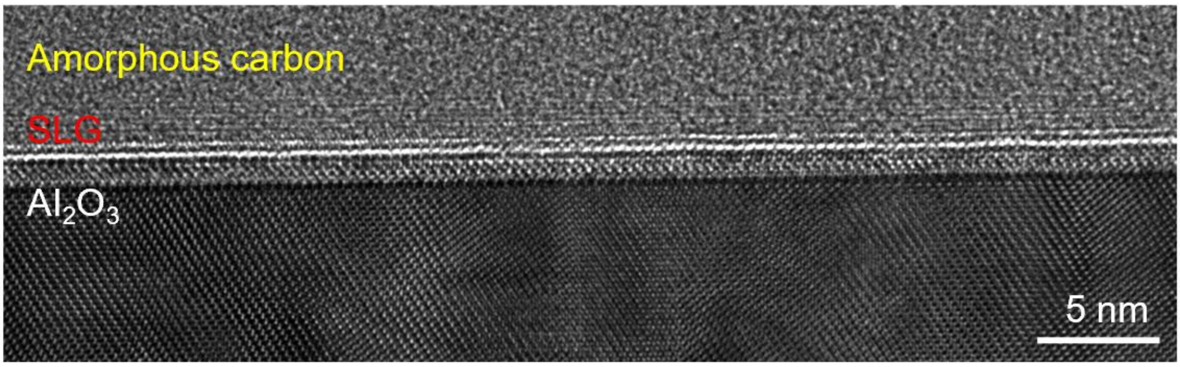

Figure S21. Cross-sectional TEM observation of the monolayer graphene/sapphire interface.

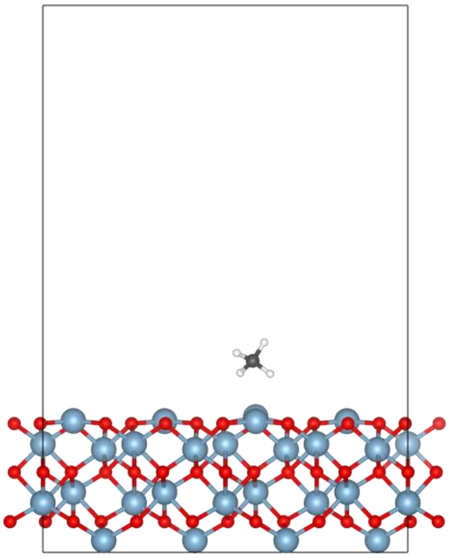

Figure S22. The optimized model employed in our AIMD calculations. Bottom two layers of atoms were fixed to represent the bulk state.

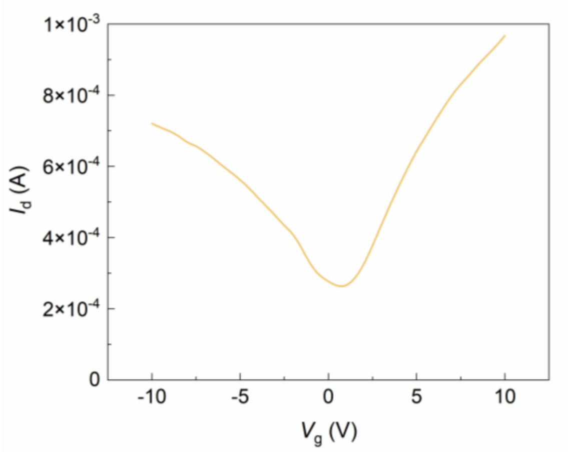

Figure S23. Transfer curve of a typical TG-GFET.

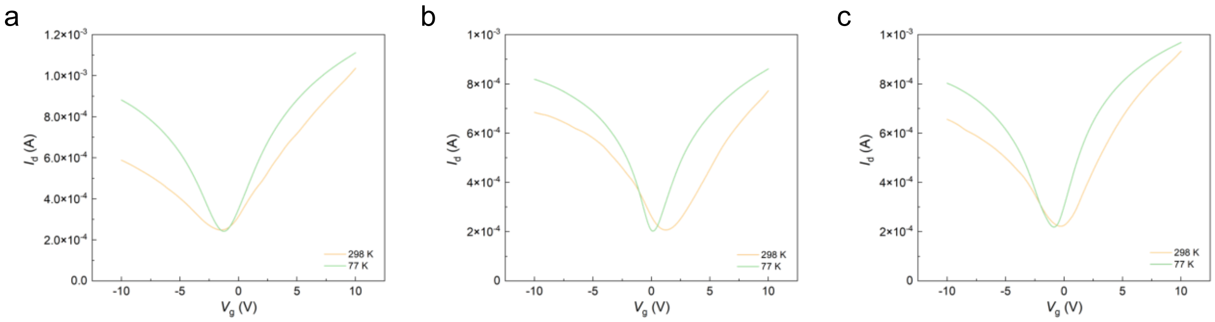

Figure S24. Transfer characteristics of three graphene devices tested at 298 K and 83 K.

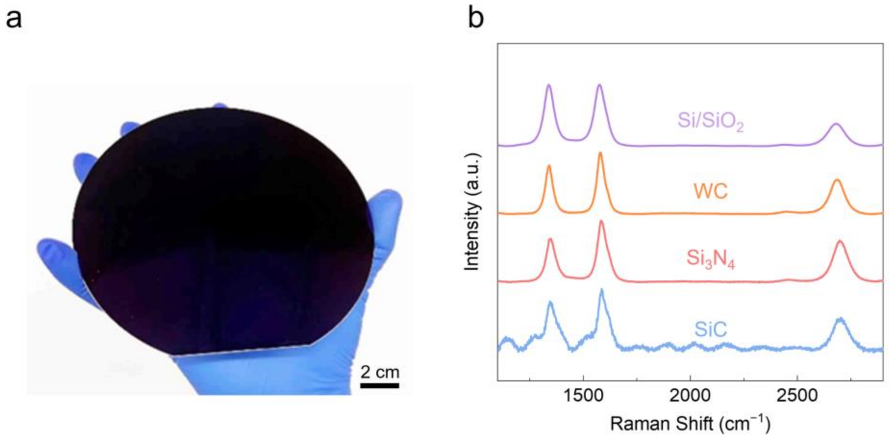

Figure S25. a) Photograph of a 6-inch graphene/Si/SiO_2 wafer. b) Raman spectra of graphene grown on Si/SiO_2, WC, Si_3N_4 or SiC substrates.

Table S1. Comparison of direct graphene growth by CVD between this work and previous reports, underscoring the advantages of this work in growth rate, wafer size, uniformity, scalability, crystallinity, and domain size.

Refs.	Substrate	Growth rate	Sample size	Uniformity	Scalability	Crystallinity	Domain size
**This work**	**sapphire**	**~30 min per batch**	**6 inch**	**I_{2D}/I_G = 1.70 ± 0.20**	**concise process, scalable in size**	**I_D/I_G< 0.10, FWHM(2D) = 31 cm^{-1}**	**~40 µm**
[7]	sapphire	~30 min per batch	2 inch	I_{2D}/I_G = 2.66 ± 0.46	substrate size limited	I_D/I_G<0.02, FWHM(2D) = 26 cm^{-1}	~40 µm
[8]	SiO_2/Si	~240 min per batch	4 inch	I_{2D}/I_G=1.22 ± 0.27	substrate size limited	I_D/I_G=0.45	~0.5 µm
[9]	quartz	~240 min per batch	60 × 60 mm^2	I_{2D}/I_G=1.18 ± 0.15	substrate size limited	I_D/I_G=0.36	~0.7 µm

Table S2. Comparison of reactor types and temperature/flow field engineering routes in representative wafer-scale CVD studies.

Refs.	Material / wafer	Reactor type	Field engineering
**This work**	**graphene on 6-inch sapphire wafer**	**cold-wall CVD**	**temperature + flow (co-field)**
[10]	MoS_2 on 12-inch Al_2O_3/Si wafer	hot-wall CVD	precursor distribution (single-field)
[11]	MoS_2 on 8-inch sapphire wafer	hot-wall CVD	precursor distribution (single-field)
[12]	graphene on 4-inch quartz wafer	hot-wall CVD	confined gas flow (single-field)
[13]	graphene on 4-inch CuNi(111) wafer	hot-wall CVD	gas velocity (single-field)
[14]	graphene on 4/6-inch sapphire wafer	cold-wall CVD	N/A (temperature/flow field not analyzed)

Table S3. Comparison of the carrier mobility of our directly grown graphene on sapphire with previously reported two-dimensional materials synthesized on typical dielectric substrates.

Refs.	Substrate	2D material type	Carrier mobility (cm^2 V^{-1} s^{-1}) at 298 K
**This work**	**sapphire**	**graphene**	**1014**
[15]	SiC	graphene	900
[16]	SiO_2/Si	graphene	531
[17]	SiO_2/Si	graphene	760
[18]	SiO_2/Si	graphene	800
[19]	sapphire	graphene	600
[11]	sapphire	MoS_2	73.2
[10]	sapphire	MoS_2	70

Supporting References

1. Kresse G, Furthmüller J. Efficient iterative schemes for ab initio total-energy calculations using a plane-wave basis set. *Phys Rev B* 1996; **54**: 11169.

2. Blöchl PE. Projector augmented-wave method. *Phys Rev B* 1994; **50**: 17953–79.

3. Perdew JP, Burke K, Ernzerhof M. Generalized gradient approximation made simple. *Phys Rev Lett* 1996; **77**: 3865.

4. Grimme S, Antony J, Ehrlich S *et al.* A consistent and accurate ab initio parametrization of density functional dispersion correction (DFT-D) for the 94 elements H-Pu. *J Chem Phys* 2010; **132**: 154104.

5. Woo TK, Margl PM, Blöchl PE *et al.* A combined car−parrinello QM/MM implementation for ab initio molecular dynamics simulations of extended systems: application to transition metal catalysis. *J Phys Chem B* 1997; **101**: 7877–80.

6. Jarzynski C. Nonequilibrium equality for free energy differences. *Phys Rev Lett* 1997; **78**: 2690–3.

7. Li J, Chen M, Samad A *et al.* Wafer-scale single-crystal monolayer graphene grown on sapphire substrate. *Nat Mater* 2022; **21**: 740–7.

8. Ci H, Chen J, Ma H *et al.* Transfer-free quasi-suspended graphene grown on a Si wafer. *Adv Mater* 2022; **34**: 2206389.

9. Liu R, Peng Z, Sun X *et al.* CO_2-promoted transfer-free growth of conformal graphene. *Nano Res* 2023; **16**: 6334–42.

10. Xia Y, Chen X, Wei J *et al.* 12-inch growth of uniform MoS_2 monolayer for integrated circuit manufacture. *Nat Mater* 2023; **22**: 1324–31.

11. Yu H, Huang L, Zhou L *et al.* Eight in. wafer-scale epitaxial monolayer MoS_2. *Adv Mater* 2024;

36: 2402855.

12. Jiang B, Zhao Q, Zhang Z *et al.* Batch synthesis of transfer-free graphene with wafer-scale uniformity. *Nano Res* 2020; **13**: 1564–70.

13. Deng B, Xin Z, Xue R *et al.* Scalable and ultrafast epitaxial growth of single-crystal graphene wafers for electrically tunable liquid-crystal microlens arrays. *Sci Bull* 2019; **64**: 659–68.

14. Mishra N, Forti S, Fabbri F *et al.* Wafer-scale synthesis of graphene on sapphire: toward fab-compatible graphene. *Small* 2019; **15**: 1904906.

15. Emtsev KV, Bostwick A, Horn K *et al.* Towards wafer-size graphene layers by atmospheric pressure graphitization of silicon carbide. *Nat Mater* 2009; **8**: 203–7.

16. Chen J, Wen Y, Guo Y *et al.* Oxygen-aided synthesis of polycrystalline graphene on silicon dioxide substrates. *J Am Chem Soc* 2011; **133**: 17548–51.

17. Pang J, Mendes RG, Wrobel PS *et al.* Self-terminating confinement approach for large-area uniform monolayer graphene directly over Si/SiO_x by chemical vapor deposition. *ACS Nano* 2017; **11**: 1946–56.

18. Kim H, Song I, Park C *et al.* Copper-vapor-assisted chemical vapor deposition for high-quality and metal-free single-layer graphene on amorphous SiO_2 substrate. *ACS Nano* 2013; **7**: 6575–82.

19. Song HJ, Son M, Park C *et al.* Large scale metal-free synthesis of graphene on sapphire and transfer-free device fabrication. *Nanoscale* 2012; **4**: 3050.
